# Uncommon Diseases of The Popliteal Artery: A Pictorial Review

**DOI:** 10.1007/s13244-016-0513-6

**Published:** 2016-08-15

**Authors:** Mohamed Jarraya, Salmi Simmons, Alik Farber, Oleg Teytelboym, Nicolas Naggara, Ali Guermazi

**Affiliations:** 1 Department of Radiology, Mercy Catholic Medical Center, 1500 Lansdowne Avenue, Darby, 19023 PA USA; 2 Division of Vascular and Endovascular Surgery, Boston University Medical Center, Boston, MA USA; 3 Department of Radiology, Musculoskeletal Unit, Boston University Medical Center, Boston, MA USA; 4 Department of Radiology, Hôpital Avicenne, AP-HP, Bobigny, France

**Keywords:** Popliteal artery, Aneurysm, Magnetic resonance imaging, Angiography, Ultrasonography

## Abstract

Uncommon diseases of the popliteal artery include cystic adventitial disease, popliteal artery entrapment syndrome (PAES) and popliteal artery aneurysm (PAA). Because all of these conditions may present with pain or intermittent claudication, imaging is crucial for differentiating them and directing management. Delayed diagnosis can lead to major complications, including acute limb ischemia. Our aim is to provide an illustrative overview of these conditions in order to make radiologists aware of them and avoid misdiagnosis for timely appropriate management.

*Teaching Points*

• *Cystic adventitial disease diagnosis is based on evidence of cysts within artery walls.*

• *A variety of anatomic variations may result in PAES.*

• *PAES may be bilateral.*

• *PAA is most commonly encountered in men.*

• *Acute complications of PAA include acute thrombosis and distal embolization.*

**Electronic supplementary material:**

The online version of this article (doi:10.1007/s13244-016-0513-6) contains supplementary material, which is available to authorized users.

## Introduction

**Fig. 1:**
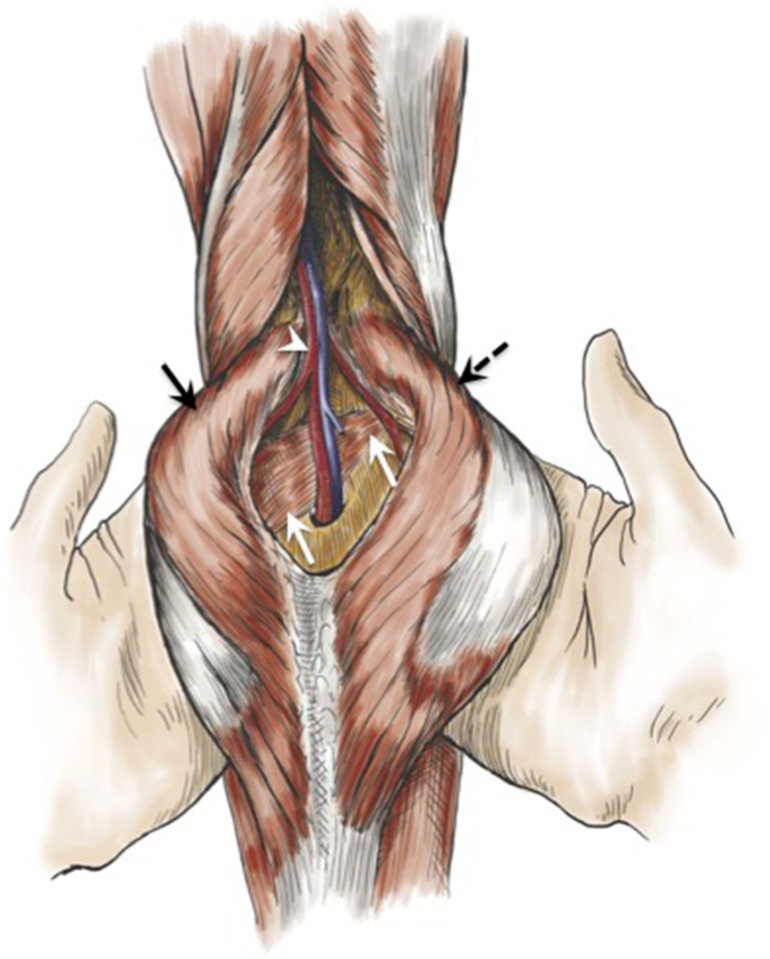
Drawing showing normal anatomy of the popliteal artery. The popliteal artery (*white arrowhead*) is shown coursing through the popliteal fossa along with the popliteal vein, posterior to the popliteus muscle (*white arrows*) and between the femoral insertions of the medial (*black solid arrow*) and lateral (*black dashed arrow*) heads of the gastrocnemius muscle

The popliteal artery is a direct continuation of the superficial femoral artery after it passes through the adductor hiatus. The normal popliteal artery and vein are located between the medial and lateral heads of the gastrocnemius muscle, posterior to the popliteus muscle (Fig. 1). Abnormalities of these relationships may result in extrinsic compression of the artery, with subsequent arterial damage and vascular insufficiency. This condition is called popliteal artery entrapment syndrome (PAES).

Microscopically, the popliteal artery is composed of three layers: intima, media and adventitia. While atherosclerotic disease involves the innermost layers of the artery [1], adventitia of the popliteal artery has a particular proclivity for an uncommon condition: cystic adventitial disease [2, 3]. The diameter of the popliteal artery is not uniform, usually larger at its midportion [4]. Popliteal artery aneurysm (PAA) is an uncommon condition, with an estimated prevalence of 0.1–1% in the general population [5, 6]. However, the presence of PAA is strongly associated with aneurysms in other locations, especially the abdominal aorta. For instance, PAA have been reported in 7–20% of patients with abdominal aortic aneurysms [6].

Although clinical symptoms resulting from these different conditions are often similar, affected patient populations are usually distinct. Radiologists should be aware of these conditions to facilitate prompt diagnosis and to appropriately direct the patient’s care. The aim of this review is to provide an illustrative overview of cystic adventitial disease, PAES and PAA.

## Cystic Adventitial Disease

**Fig. 2:**
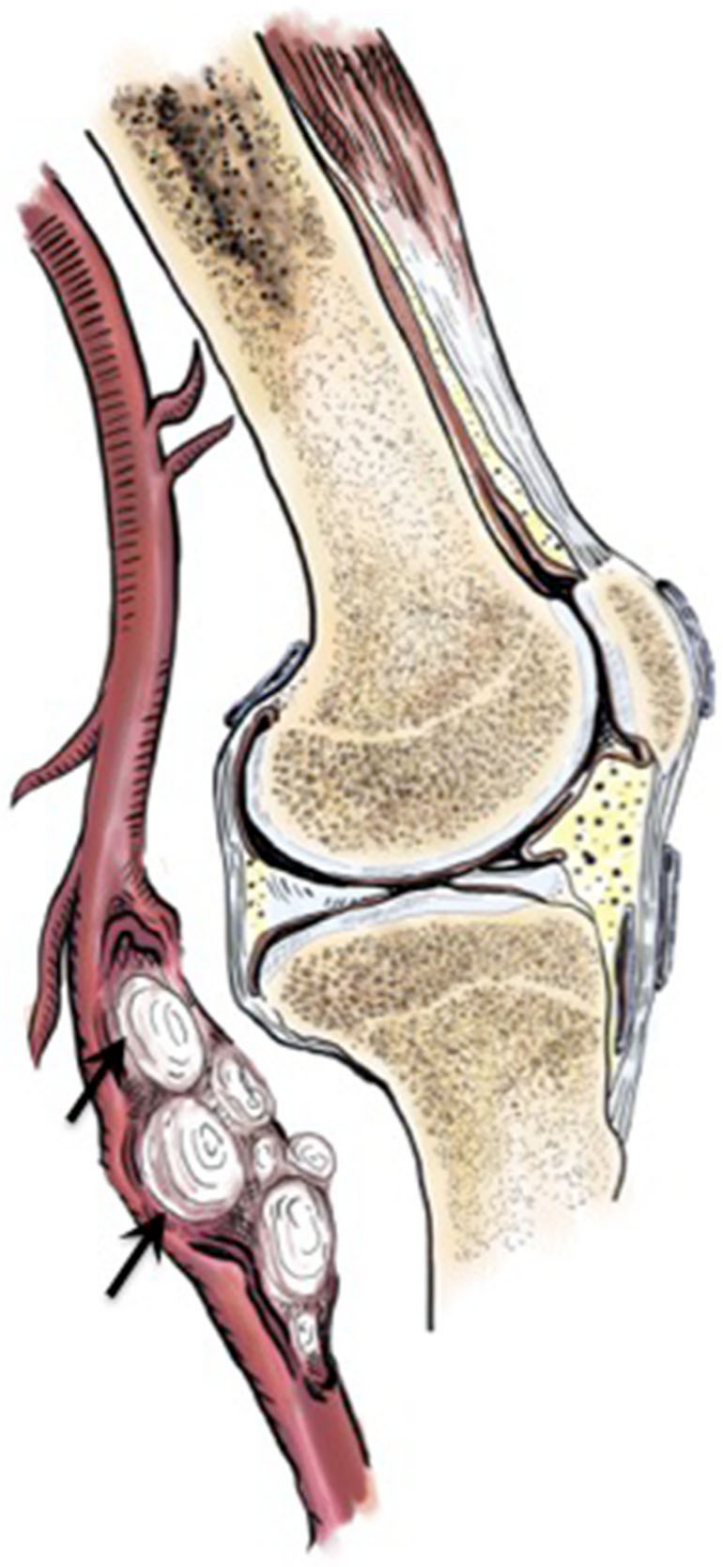
Drawing showing cystic adventitial disease of the popliteal artery. Eccentric cystic lesions arising from the anterior wall of the popliteal artery are shown (*arrows*)

**Fig. 3:**
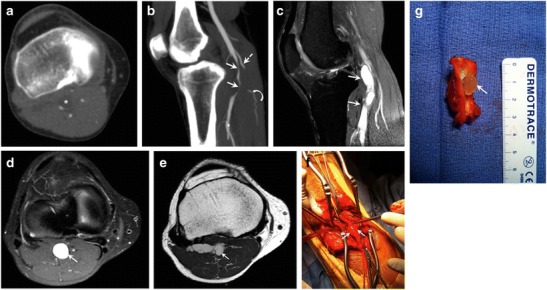
Adventitial cystic disease of the popliteal artery in a 50-year-old woman with intermittent claudication of the right lower extremity. (**a**) Axial CT angiography shows a well-circumscribed fluid-density lesion within the popliteal fossa (*asterisk*) compressing the popliteal artery. (**b**) Sagittal maximum-intensity projection reconstruction of CT angiography shows eccentric stenosis of the popliteal artery (scimitar appearance), with significant narrowing of the vessel lumen (*solid arrows*). Note the collateralization (*curved arrow*) with dilation of the sural artery (*dashed arrow*). (**c**, **d**) Sagittal and axial fat-suppressed T2-weighted MR images show hyperintense signal of the arterial wall mass (*arrows*) confirming its cystic nature. (**e**) Axial T1-weighted MR image shows hyperintense signal of the cystic mass (*arrow*) related to mucinous content. (**f**) Intraoperative photo showing cystic lesion arising from the popliteal artery wall (*arrows*). (**g**) Photograph of the resected specimen after longitudinal transection, showing cystic lesion developing within the lumen of the popliteal artery (arrow)

Cystic adventitial disease is an uncommon condition in which mucinous material containing varying combinations of mucopolysaccharides and mucoproteins form within the adventitia of arteries and veins [7]. While any peripheral artery or vein may be affected, the disorder has a striking proclivity for the popliteal artery (89% of cases) (Figs. 2 and 3) [8]. This disorder typically affects young to middle-aged men without cardiovascular risk factors. The male-to-female ratio is approximately 4.6:1 [7]. The pathogenesis of cystic adventitial artery disease is controversial. Proposed hypotheses include:

- Micro-traumatic: supported by the predominance of disease among middle-aged men with heavy occupational activity involving the legs, and some reports of definite trauma prior to discovering the cyst [9]. Repeat stretch injuries are thought to result in cystic degeneration [7].
- Developmental: from embryological migration of mucin-secreting cells from the knee joint to the artery’s adventitia. In this case, mucin-secreting cells from the endothelium of the knee joint appear in the adventitia of the artery, leading to the development of tense cysts over several years [7]. However, this theory implies that the adventitial cyst content would be characterized by epithelial secretion rather than collagen and ground substance breakdown [7].
- Synovial: from ectopic synovial ganglions migrating along the vascular branches from the adjacent knee joint capsule [8]. This is supported by reported coexistence of arterial adventitial cyst and adjacent periarticular tendinous ganglia, with radiologic and surgical reports showing a connection between these cysts or between the adventitial cyst and the joint itself [10, 11]. A recent review on cystic adventitial disease reported joint connection with arterial wall cysts in 13% of reported cases, regardless of the affected artery [8]. The synovial theory is favored over the developmental theory by the chemical nature of the cystic content: collagen and ground substance breakdown products.

The most common clinical presentation is intermittent claudication (89% of cases), although critical acute and chronic limb ischemia have been described in up to 7% of cases [8].

Imaging plays a central role in the diagnosis of cystic adventitial disease. While all modalities are helpful, ultrasound, magnetic resonance (MR) imaging, and MR angiography are the most helpful, because of their ability to identify cystic lesions within the wall of the popliteal artery [8]. Ultrasound shows anechoic or hypoechoic round lesions with well-defined margins and posterior acoustic enhancement, originating from the arterial wall. These lesions may be multilocular, with thin septa, and may show low-level echoes due to gelatinous content [2]. Doppler ultrasound study shows increased peak systolic velocities based on the degree of stenosis [8].

MR imaging shows multilocular cystic masses within the arterial wall. Fluid-like hyperintensity on fluid-sensitive sequences such as short tau inversion recovery (STIR) and spectral presaturation with inversion recovery (SPIR) favors the cystic nature of the lesion. Due to the mucinous content of the cysts, T1-weighted images may also show signal hyperintensity [8]. Three-dimensional acquisition is recommended to help visualize the connection between the cystic component and the knee joint [10]. MR angiography shows extrinsic arterial compression with smooth margins [3]. Arterial stenosis may appear scimitar-shaped in the case of eccentric stenosis, and hourglass-shaped when the cystic lesions are concentric [7]. To our knowledge, there is no report on MR arthrography in cystic adventitial disease.

Computed tomography (CT) findings include a circumscribed fluid-density mass compressing the artery, but often without the multilocular appearance, because of lower tissue resolution than with MR imaging [8]. Cystic adventitial disease was initially described on conventional angiography [7], but with the advent of cross-sectional imaging, it has become the least useful modality, as it only shows luminal stenosis.

Treatment may be resectional or non-resectional. Complete resection of the affected arterial segment is followed by end-to-end anastomosis, repair using a patch, or reconstruction by interposition of a vein, homograft, or prosthetic graft. Alternatively, non-resectional techniques include imaging-guided percutaneous cyst aspiration, open cyst aspiration, and percutaneous transluminal angioplasty with cyst evacuation [8]. The exact recurrence rate is unknown, due to rarity of the disease [8]. Previous reports on imaging-guided cyst aspiration, including ultrasound-guided aspiration and angioplasty, have shown both sustained remission and rapid progression of the cystic adventitial disease [12–14]. The initial success rate of image-guided percutaneous cyst aspiration is reported to be much lower than that of surgery [15].

## Popliteal Artery Entrapment Syndrome

**Fig. 4:**
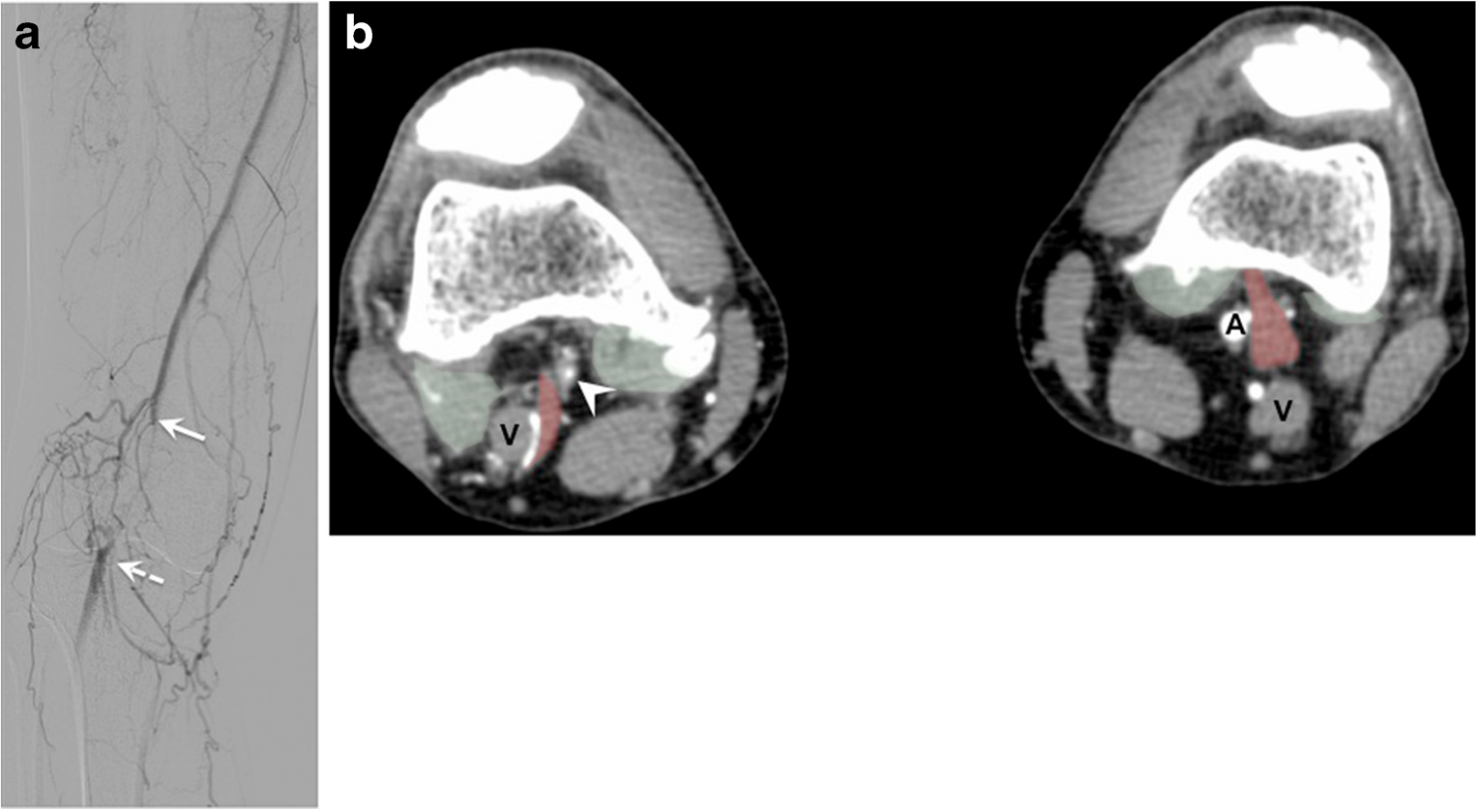
Popliteal artery entrapment syndrome (PAES) in a 52-year-old man with a history of right calf claudication for 2 years. (**a**) Digital subtraction angiography shows abrupt occlusion of the proximal popliteal artery (*solid arrow*), which reconstitutes at its midportion (*dashed arrow*). A cine sequence is presented in Movie 1. PAES was suspected and patient underwent CT angiography. (**b**) Axial images of CT angiography reveal severe narrowing of the right popliteal artery (*arrowhead*), with an abnormal muscle slip (*red transparent overlap*) originating from the medial head of the gastrocnemius muscle and coursing between the popliteal vessels (V = popliteal vein). The contralateral side shows a similar appearance of abnormal muscle slip (*red transparent overlap*); however, the left popliteal artery is patent (A). *Bilateral green transparent areas* correspond to normal insertions of the gastrocnemius insertions

PAES is an uncommon entity, reported in 3.5% of autopsy cases [16], which results from abnormal relationship of the popliteal artery with neighbouring musculotendinous structures. Arterial compression may result in chronic vascular microtrauma with local premature arteriosclerosis and thrombus formation [17]. PAES is a progressive condition, for which early diagnosis is warranted to prevent serious complications, including post-stenotic aneurysms and distal embolization, with risk of subsequent ischemia [17, 18]. PAES can be bilateral [19], and therefore screening of the contralateral knee is warranted in case of PAES (Fig. 4).

Similarly to cystic adventitial disease, PAES should be considered in young adults with no cardiovascular risk factors presenting with intermittent claudication for several months or years [19]. Two critical moments during embryogenesis can explain PAES: formation of the popliteal artery and migration of the gastrocnemius muscle [20, 21]:

1. While the embryonic popliteal artery, which is derived from the axial artery, is located anterior to popliteus muscle, it regresses progressively and is replaced by the definitive popliteal artery posterior to the popliteus muscle by the seventh week. Failure of regression of the embryonic popliteal artery results in entrapment by the popliteus muscle.
2. Prior to formation of the definitive popliteal artery, the primitive gastrocnemius muscle located laterally divides into the lateral gastrocnemius, which remains inserted on the lateral femoral condyle, and the medial gastrocnemius. The latter migrates until definitively inserted in the medial femoral condyle. Under normal circumstances, medial gastrocnemius migration occurs around the sixth week, before formation of the definitive popliteal artery. Delayed migration (or early formation of the definitive popliteal artery) results in capture and entrapment of the definitive popliteal artery during the migration of the medial gastrocnemius from the lateral to medial position, causing medial displacement of the popliteal artery.

**Fig. 5:**
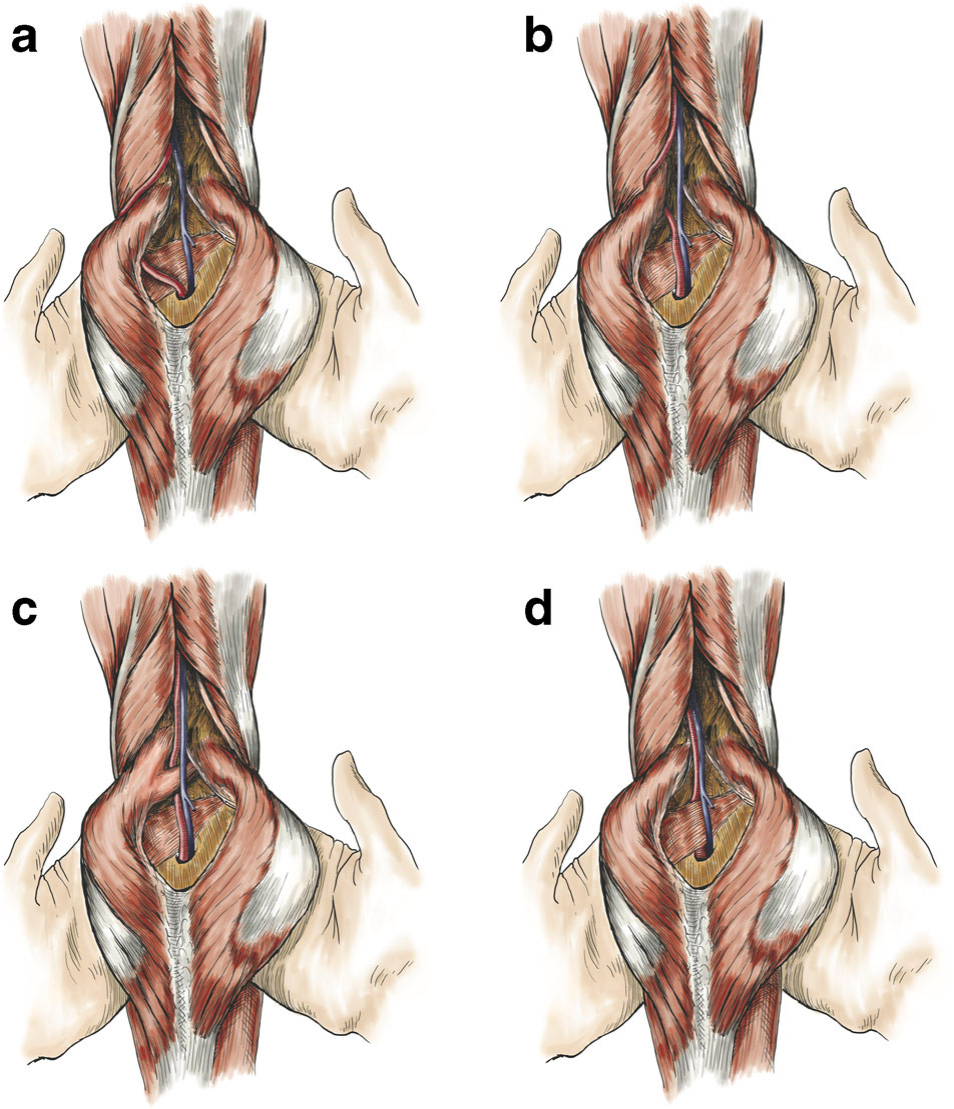
Drawings illustrate the classification scheme for PAES. (**a**) Type 1: the medial head of the gastrocnemius muscle has normal insertion, displacing the popliteal artery around and beneath the muscle. (**b**) Type II: the medial head of the gastrocnemius arises from an abnormal position, displacing the popliteal artery medially. (**c**) Type III: entrapment is caused by an abnormal slip of the gastrocnemius muscle. (**d**) Type IV: entrapment is caused by a fibrous band or by the popliteal muscle

A commonly used classification for PAES was reported by Delaney et al. (Fig. 5) [22, 23]:

Type 1 The popliteal artery is abnormally running medial to the medial head of the gastrocnemius, which has a normal insertion.
Type 2 Entrapment results from an aberrant insertion of the medial head of the gastrocnemius muscle.
Type 3 Entrapment results from an accessory slip from the medial head of the gastrocnemius muscle.
Type 4 The popliteal artery is compressed when passing under the popliteal muscle or under a fibrous web.

**Fig. 6:**
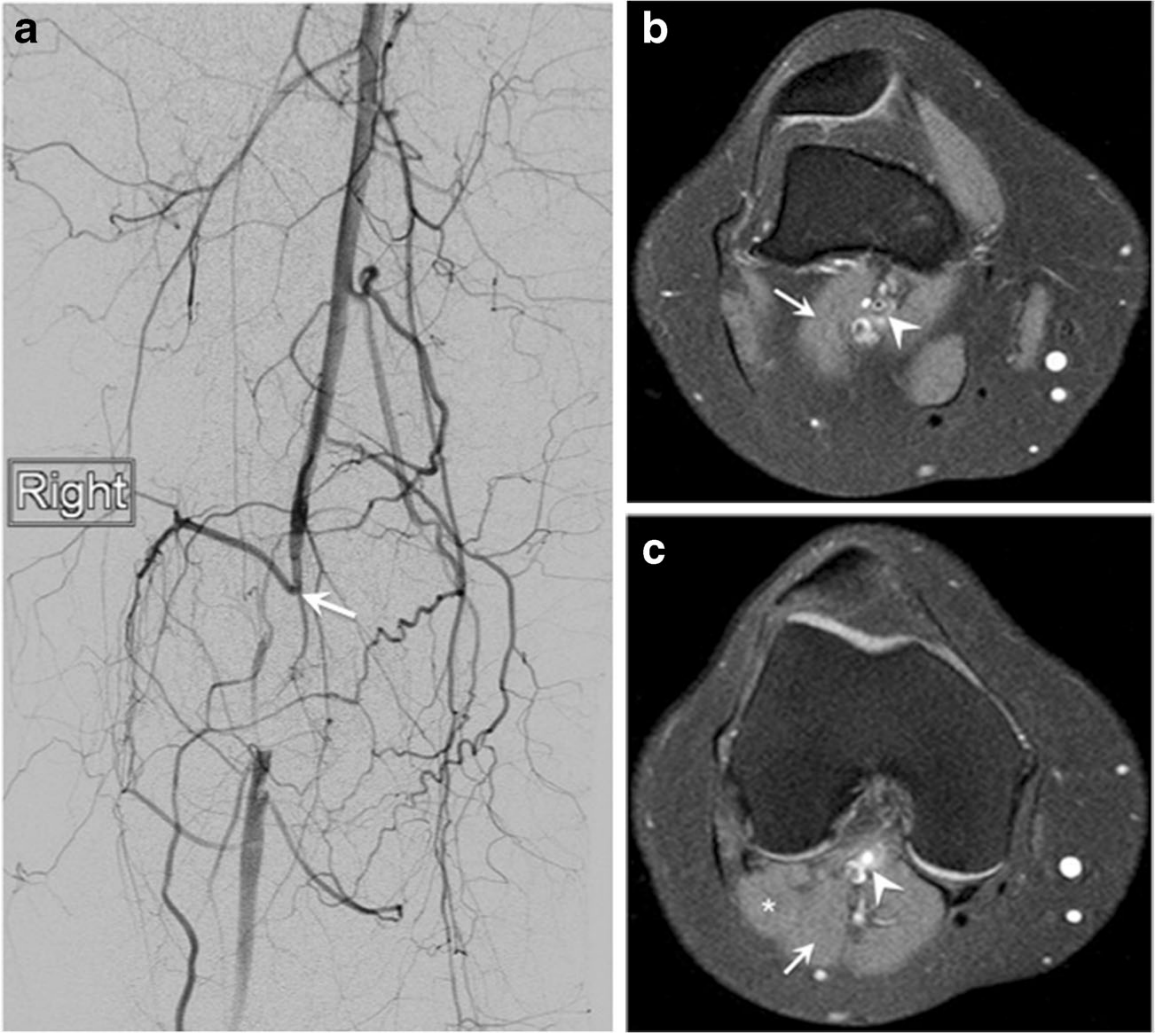
Popliteal artery entrapment syndrome (PAES) in a 31-year-old woman admitted to the hospital because of a 6-week history of right calf pain and numbness. Patient also reported half-block claudication. (**a**) Lower extremity digital subtraction angiography reveals obliteration of the popliteal artery (*arrow*). Given the lack of risk factors for an arterial thrombus, an anatomic etiology was suspected. (**b**, **c**) Axial selective partial inversion recovery (SPIR) MR images of the right knee showing an accessory slip of the lateral head of the gastrocnemius muscle (*arrow*) crossing anterolateral to the popliteal vessels, with resulting compression of the popliteal artery (*arrowhead*). The accessory slip (*arrow*) joins the lateral head of the gastrocnemius distally (*star*). This finding supports a diagnosis of PAES

**Fig. 7:**
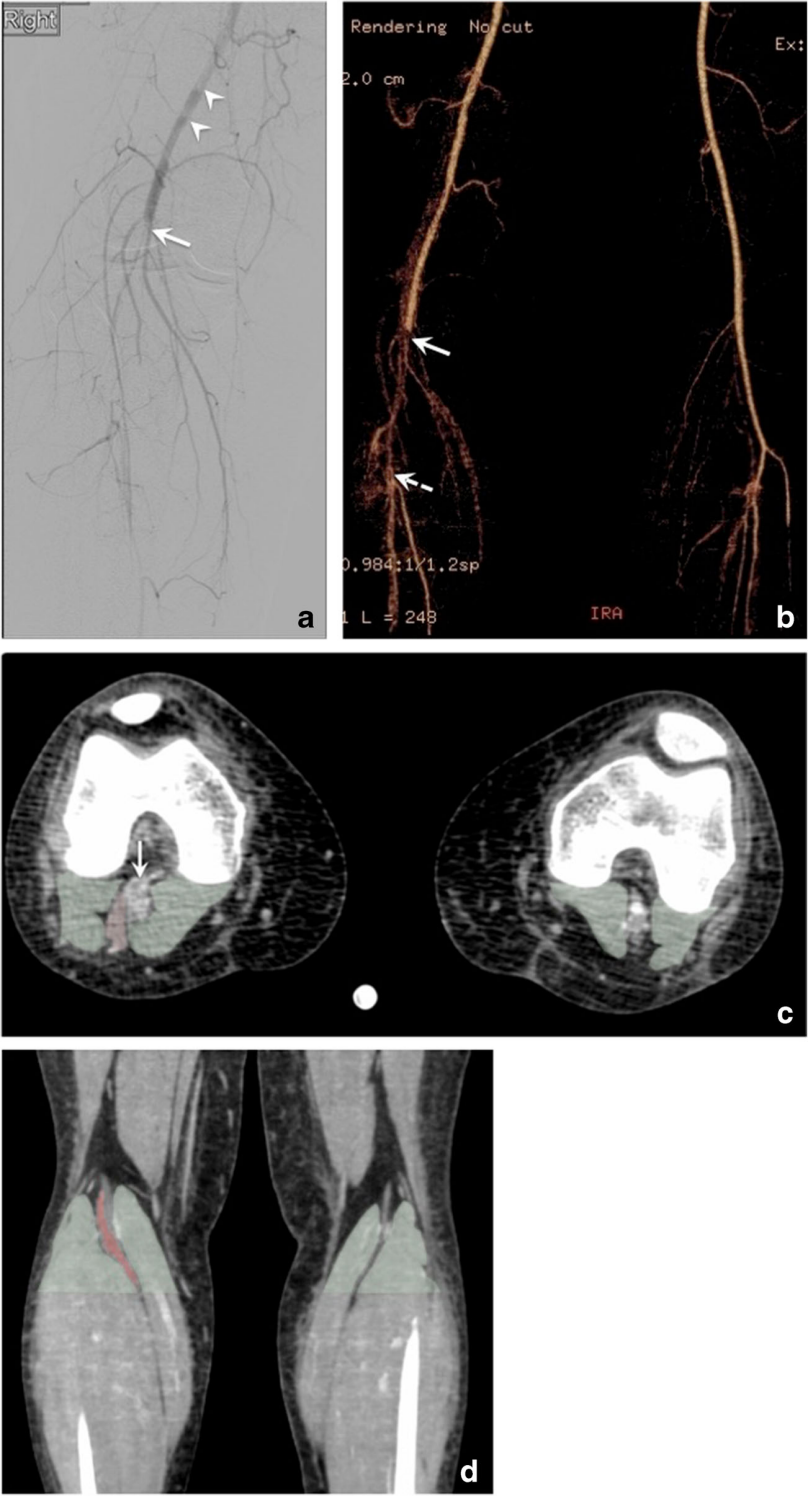
Popliteal artery entrapment syndrome (PAES) in a 38-year-old woman with a 4-week history of right foot pain, admitted to the hospital for gangrenous fourth toe. (**a**) Digital subtraction angiogram shows total occlusion of the right popliteal artery (*arrow*), with filling defects of the distal superficial femoral artery (*arrowheads*) consistent with thrombus, and multiple collaterals around the knee. PAES was suspected and patient underwent CT angiogram. (**b**) Maximum-intensity projection reconstruction from lower extremity CT angiogram demonstrated total occlusion of the right popliteal artery (*arrow*) extending into the proximal anterior tibial artery, with distal reconstitution (*dashed arrow*). (**c**) Axial CT angiography image shows occlusion of the right popliteal artery (*arrow*), and an extra slip of muscle lateral to it (*red transparent overlap*). No comparable structure is seen on the left side, which suggests right PAES. *Green transparent overlapping areas* of the normal gastrocnemius heads bilaterally are added to display the asymmetrical appearance between right and left knees. (**d**) Coronal reconstruction of CT angiography showing the gastrocnemius heads (*green transparent overlap*) with an extra slip of muscle (*red transparent overlap*) causing asymmetric appearance, and possibly originating from the lateral head

Although not included in this classification, the lateral head of the gastrocnemius muscle may also have an aberrant origin, resulting in compromise of the popliteal artery (Figs. 6 and 7) [24, 25].

While PAES is defined by an abnormal relationship between the popliteal vessel and neighboring musculotendinous structures, a more frequent entity, functional PAES. results from muscle solicitation rather than structural abnormality. It occurs in young athletic individuals, particularly in performance athletes. Compression of the popliteal artery may be caused by the gastrocnemius muscle (above the knee) or plantaris muscle (below the knee). Dynamic testing is necessary for diagnosis. Depending on the imaging modality, this testing may include repeat dorsiflexion and plantar flexion, without and with resistance, and may even involve assessment of the patient in the erect position with plantar flexion by standing on the toes, with repeated plantar flexion until the patient is symptomatic. Evidence of both leg pain and reduction in the diameter of the popliteal artery are required for positive diagnosis of functional PAES [20, 26]. Ultrasound is the modality of choice for this functional testing, given its inherent adaptability for dynamic imaging. CT and MR angiography require sustained muscle solicitation during image acquisition [20]. MR angiography is preferable because of the lack of ionizing radiation, and has been shown to adequately detect popliteal artery compression when scanning is performed in either a neutral position or with active plantar flexion [27, 28]. Dynamic testing requires a system that maintains the patient’s foot in the proper position and against which forced active plantar ankle flexion can be performed for up to 30 s [20, 28].

Imaging plays a central role in diagnosing PAES. In addition to arterial displacement, ultrasound may show the presence of a fibrous band between the popliteal artery and vein. Popliteal stenosis, post-stenotic dilation, and thrombus may be seen in the case of long-standing disease. Diagnosis of functional PAES relies upon evidence of arterial diameter reduction and increased maximum systolic velocity at the site of compression upon dynamic testing.

Conventional angiography is the classical modality for screening and diagnosis of PAES, showing medial displacement and compression of the artery as well as irregularities of the vessel wall in an otherwise normal arterial tree. Other findings are related to complications of long-standing disease, such as pre- and post-stenotic dilation, aneurysm, and thrombosis [19, 29]. However, no information on the abnormal musculotendinous relationship with the popliteal vessel can be obtained with conventional angiography (Movie 1).

MR and CT angiography offer precise analysis of the muscle course and potential conflict with the popliteal vessels. Findings include medial deviation of the vessel by an abnormal slip of the medial or lateral head of the gastrocnemius muscle (Figs. 4, 6, and 7) (Movies 2 and 3) or entrapment of the popliteal artery by a fibrous band [20].

Treatment consists of releasing the trapped vessel by myomectomy. While lateral transposition of the popliteal artery is always needed, the presence of vascular complications such as stenosis, aneurysm, or occlusion warrants additional vascular reconstruction by endarterectomy or bypass grafting [30]. Endovascular treatments are not effective in PAES and are associated with high rates of reocclusion unless the underlying cause of entrapment is treated [18].

## Popliteal Artery Aneurysm

**Fig. 8:**
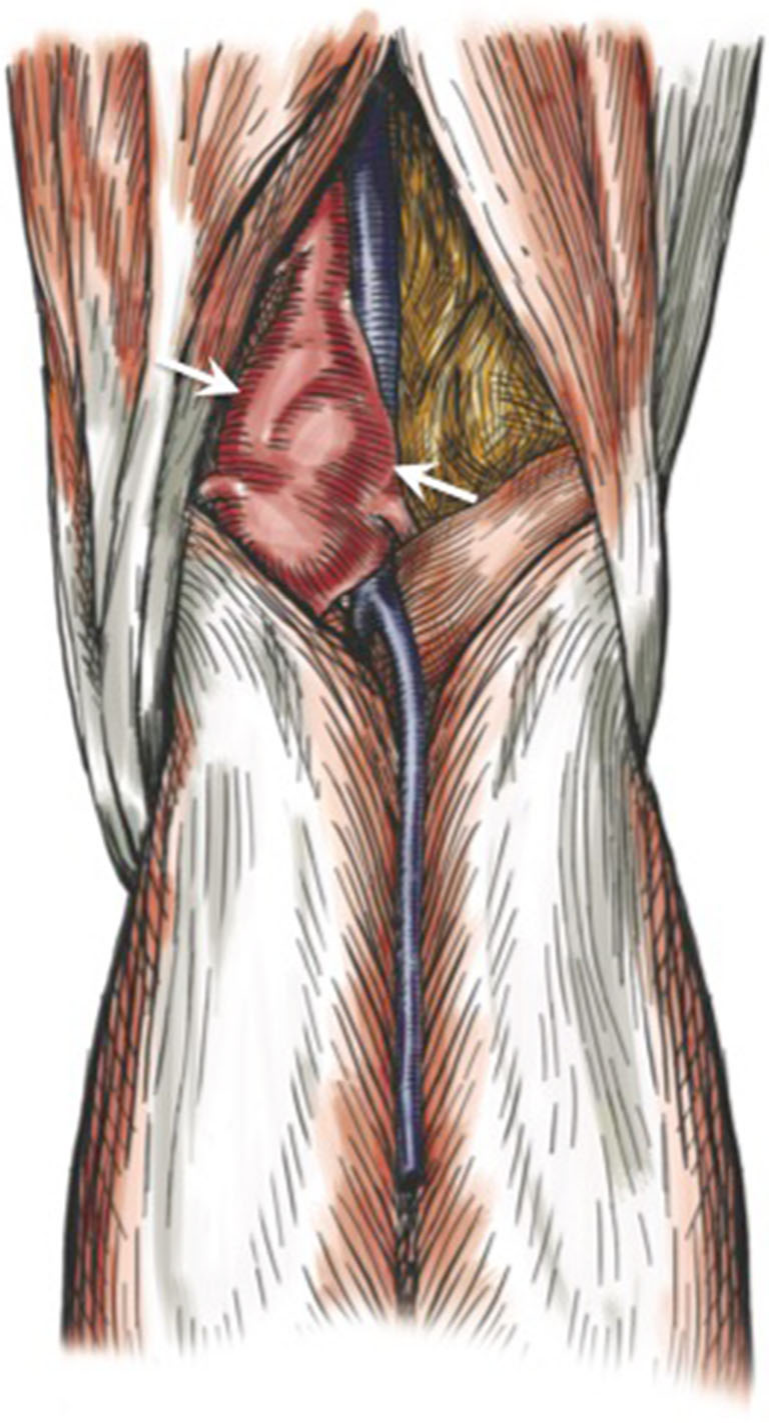
Drawing illustrating a fusiform aneurysm of the popliteal artery (arrows)

There is no agreed-upon definition for PAA. Some authors define it as having a diameter exceeding 0.7cm [23], while others report a threshold diameter of 2 cm, or 150% of the normal arterial diameter [31, 32]. PAA may be saccular or fusiform in configuration (Fig. 8). Atherosclerosis is the most common underlying etiology. PAA is the most common peripheral arterial aneurysm, accounting for 70% of lower extremity aneurysms [23, 31]. They are bilateral in 50–70% of cases, and are encountered almost exclusively in men, with women representing only 3–5% of cases [31, 33, 34]. PAA are mainly encountered in the sixth and seventh decade of life [23]. Patients diagnosed with PAA should be scanned for contralateral PAA and abdominal aortic aneurysms, because of strong reported association [23]. For instance, PAA have been reported in 7–20% of patients with abdominal aortic aneurysm [6].

**Fig. 9:**
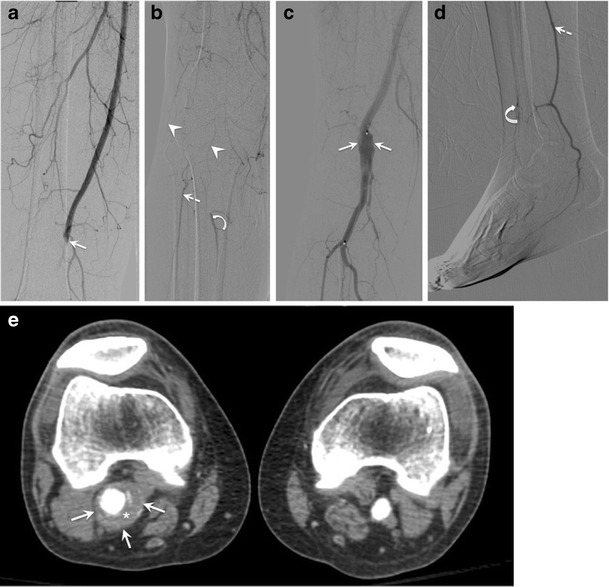
Popliteal artery aneurysm (PAA) in a 52-year-old man admitted for cold right foot. (**a**, **b**) Digital subtraction angiography on admission shows abrupt occlusion of the proximal popliteal artery at the level of the adductor hiatus (*solid arrow*), with minimal collateral vessels around the knee (*arrowheads*), suggesting acute thrombosis. Distally, there is revascularization of the peroneal (*dashed arrow*) and anterior tibial (*curved arrow*) arteries. Patient underwent intravascular catheter-directed thrombolysis, and lytic check at 12 h (not shown) showed incomplete lysis of the thrombus. Patient was continued on the same treatment. (**c**, **d**) Digital subtraction angiography at 22 h post-lysis reveals a large PAA (*solid arrows*), with re-permeabilization of the posterior tibial artery (*dashed arrows*). However, the distal anterior tibial artery is barely opacified, suggesting vascular changes from chronic distal embolization resulting in tissue loss. (**e**) Axial CT angiography shows a larger PAA than expected from angiography images (*solid arrows*), with mural thrombus (*asterisk*). These findings characterize classical acute (acute thrombosis) and chronic (loss of distal run-off from repeated distal embolization) complications from PAA

PAA have an insidious presentation, often with ischemia as a first manifestation. The most feared complication is an acute aneurysm thrombosis, which may lead to irreversible leg ischemia or viable ischemia, both requiring immediate management. Distal embolization often goes unnoticed, causing a loss of distal run-off prior to diagnosis (Fig. 9). Rupture, however, is a rare complication (<5%) [31]. If left untreated, complications occur in up to 31% of cases [35].

Ultrasound can establish the diagnosis and patency of PAA. Conventional angiography may show arterial dilation or occlusion. Cross-sectional imaging, on the other hand, can delineate the aneurysmal sac and detect mural thrombus [23] (Fig. 9).

The main points of controversy in the management of PAA are (1) when to carry out repair of asymptomatic PAA, and (2) what procedure to perform in the case of acute aneurysm thrombosis. Because of its insidious evolution, repair of PAA is recommended if there are no surgical contraindications [31]. PAA repair can be performed by open surgery, typically a bypass with aneurysm exclusion, or endovascular therapy, typically deployment of a self-expanding stent graft, depending on the surgeon’s preference, as there is not sufficient long-term data for comparison [31]. In the case of aneurysm thrombosis, thrombolytic therapy may be effective for achieving recanalization of the distal popliteal artery, especially in patients who can withstand an additional period of ischemia (Fig. 9) [36]. However, the success of thrombolytic therapy may be limited in cases of organized thrombus in the wall of the PAA, and may also result in distal embolization of thrombus fragments.

## Summary

**Fig. 10:**
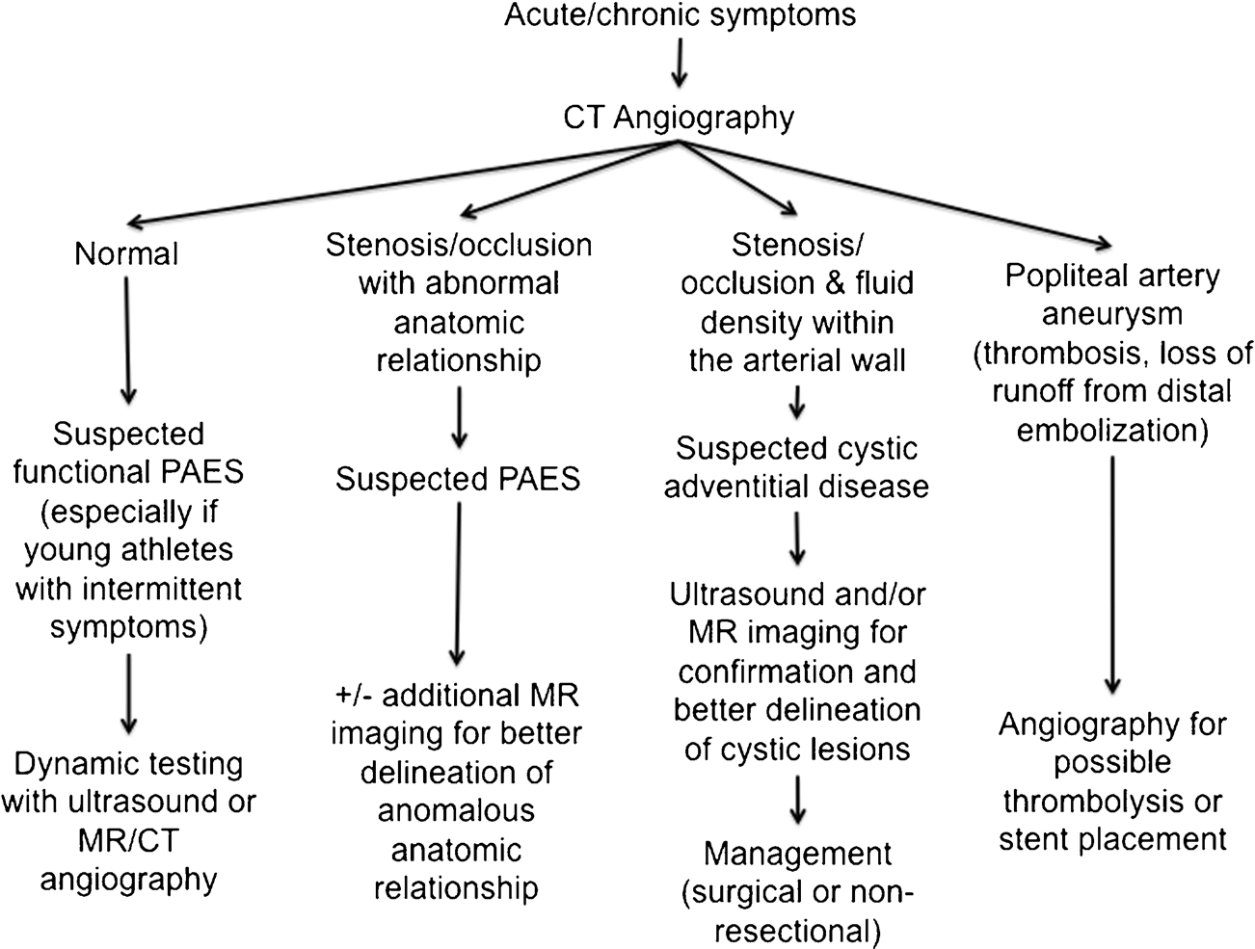
Flowchart showing suggested imaging modalities for each condition based on CT angiography findings

We have reviewed uncommon conditions affecting the popliteal artery. An algorithm for suggested imaging workup is presented in Fig. 10. For both adventitial cystic disease and PAES, CT angiography and MR angiography play a key role in the timely diagnosis of these conditions, thanks to their high spatial and contrast resolution. Ultrasound has proven to be particularly useful for dynamic testing in establishing a diagnosis of functional PAES. Although often asymptomatic, PAA have an insidious evolution, leading to progressive tissue loss from distal embolization or thrombosis. Surgical repair is recommended even when PAA is asymptomatic.

## Electronic supplementary material

Below is the link to the electronic supplementary material.

Movie 1 Cine sequence of digital subtraction angiography of the same patient as Fig. 4, showing abrupt occlusion of the proximal popliteal artery, with revascularization at its midportion. (MP4 416 kb)

Movie 2 Popliteal artery entrapment syndrome. Cine sequence of coronal proton density-weighted MR images in a 39-year-old man with history of left calf intermittent claudication. Medial displacement of the popliteal artery (A) caused by an anomalous origin of the medial gastrocnemius muscle (*yellow arrow*). Normal course of the popliteal artery is along its vein (V). (MOV 3423 kb)

Movie 3 Same patient as movie 2. Cine sequence of axial CT images shows abnormally high origin of the medial head of left gastrocnemius muscle (*red transparent overlap*) causing medial displacement and occlusion of the popliteal artery. The medial head of the right gastrocnemius muscle (*green transparent overlap*) has normal insertion and relationship with popliteal artery. (MP4 460 kb)
